# Two rare cases of severe community-acquired bloodstream infections: a clinical case report

**DOI:** 10.3389/fimmu.2024.1364391

**Published:** 2024-12-10

**Authors:** Li-Na Meng, Gang Li

**Affiliations:** Department of Intensive Care Unit, Peking University International Hospital, Beijing, China

**Keywords:** community-acquired bloodstream infections, metagenomics, next-generation sequencing, case report, *Klebsiella pneumoniae*

## Abstract

**Background:**

The escalating demographic shift towards an aging population and the widespread occurrence of immunological diseases have contributed to an elevation in the frequency of community-acquired infections. Notably, among these infections, community-acquired bloodstream infections (CABSI) stand out due to their significant lethality. Detailed medical history inquiries, assessment of underlying immune status, detection of the source of infection, and initial precise identification and treatment of the infectious agents can improve the prognosis of CABSI.

**Case description:**

In this paper, two incidences of severe CABSI with insidious onset and rapid progression are described. Both patients had compromised basic immunity: one developed the infection following unhygienic dietary practices, and the other after repeated enemas leading to intestinal damage. Blood genomic sequencing revealed the presence of *Klebsiella pneumoniae* and *Staphylococcus aureus* in the respective cases, with the origin of the infection traced back to the gastrointestinal tract. Both patients experienced positive outcomes following targeted antibiotic therapy, fluid resuscitation, support for organ function, and surgical interventions. Nevertheless, one patient manifested dry gangrene in the extremities during the course of treatment, potentially associated with the administration of vasoconstrictor drugs, considering the compromised baseline vascular conditions.

**Conclusion:**

Clinicians are advised to expeditiously uncover concealed medical histories and potential sources of infection in patients, thoroughly investigate the origin of the infection, and initiate early genomic testing to ascertain the specific nature of the infection. This proactive approach aims to facilitate precise treatment strategies and, consequently, enhance the overall prognosis.

## Introduction

Bloodstream infections (BSI) have become a major cause of fatality among critically ill patients worldwide, ranking among the top seven causes of fatalities in North America and Europe. Approximately 7.8% of patients with BSI may develop sepsis and septic shock. The escalating prevalence of community-acquired bloodstream infections (CABSI) has emerged as a noteworthy concern, potentially associated with the growing older adult demographic, immunocompromised states arising from chronic illnesses, and the intricate nature of medical care within community settings (1). Prevention and early detection of CABSI, along with timely initiation of resuscitative treatment measures, pose a significant challenge and are essential to improve outcomes (2). Prior research indicates that early comprehensive testing conducted in emergency departments can mitigate the risk of patients progressing to sepsis (3–5). Reports indicate that about 40% of patients diagnosed with CABSI admitted through emergency departments succumb within 3 days of hospitalization, and 80% within 7 days (6). Early identification of risk factors and determination of the origin and characteristics of the infection following hospital admission are crucial for subsequent treatment. The predominant pathogens in CABSI are Enterobacteriaceae, especially *Escherichia coli* and *Klebsiella pneumoniae*. In recent years, there has been an increasing trend in infections caused by *Staphylococcus aureus*, accounting for approximately 10–15% of bloodstream infections. Half of these patients exhibit migratory lesions, manifesting as multiple lung infiltrates, liver abscesses, purulent meningitis, skin abscesses, and other conditions (7). Primary bloodstream infections are challenging to identify due to the absence of a clear site of infection. Notably, for Enterobacteriaceae,colonization or environmental exposure can lead to primary or secondary bloodstream infections (8). According to reports, cases of blood-stream infections caused by bacteria originating in the gastrointestinal tract, accounts for about 7–22% of Gram-negative bloodstream infections in the intensive care unit (ICU) (9). Although the antibiotic resistance rates in CABSI are lower than hospital-acquired bloodstream infections (HABSI). There is still a more severe challenge in the treatment of CABSI (10). Shortening the pre-hospital consultation time, early detection, and appropriate initial empirical antibiotic treatment are associated with reduced mortality, improved antibiotic management, and shorter hospital stays (11, 12). With the characteristics of short time, high sensitivity and specificity, the next-generation sequencing technology of metagenome is attracting more and more attention from clinicians. Some literatures have proposed that the role of mNGS in the diagnosis of infectious diseases is becoming increasingly mature (13). MNGS can directly identify rare, novel, difficult to detect, and mixed infection pathogens from clinical samples, and has shown great potential in predicting drug resistance. It can be used to guide clinical anti infection strategies and promote rational drug use. There are reports that the sensitivity, specificity, positive predictive value, and negative predictive value of dual mNGS (plasma cfDNA and blood cells) in bloodstream infections with combined neutropenia are 95.2%, 94.6%, 95.2%, and 94.6%, respectively (14). However, due to operational and cost issues, mNGS is still considered the ultimate solution to clinical infection problems, delaying the optimal diagnosis and treatment timing. Clinical doctors need to have a comprehensive understanding of the functions and limitations of this method to ensure its timely and correct application in clinical diagnosis.

## Case introduction

**Figure 1:**
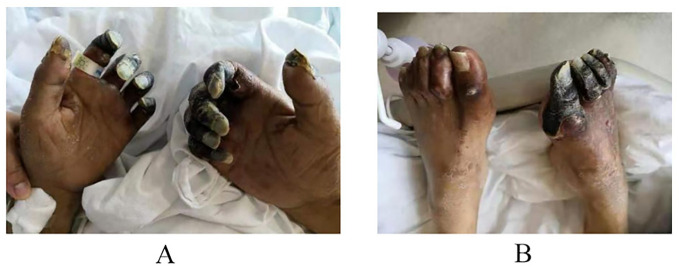
As the patient has suffered from diabetes for many years and has poor vascular conditions, dry gangrene occurs at the extremities after the use of moderate dose of norepinephrine.

Patient-1: A 60-year-old male patient, presented to the hospital with a two day history of vomiting and fever following consumption of rice noodles. The patient scored 22 on the APACHE II scale. He had a 20-year history of diabetes mellitus, which was not regularly monitored or treated, as indicated by a glaciated hemoglobin level of 13.6%. He also had a history of cerebral infarction. Upon presentation to the emergency department, the patient manifested symptoms including elevated fever (T 39.8°C), altered consciousness, a respiratory rate ranging from 30 to 40 breaths per minute, blood pressure measuring 85/40 mmHg, anuria persisting for 6 hours, and arterial blood gas analysis revealing metabolic acidosis and hypoxemia. CT scans of the chest and abdominopelvic region revealed no clear source of infection. The patient underwent endotracheal intubation and initial fluid resuscitation in the emergency department before being shifted to the ICU. Prior to ICU admission, comprehensive tests including complete blood count, white blood cells (WBC 21.26×10⁹/L),C-reactive protein (CRP 220mg/L), procalcitonin (PCT>100ng/ml), interleukin-6(IL-6 534pg/ml), bronchoalveolar lavage fluid, and blood bacterial culture, along with metagenomic next-generation sequencing (mNGS), were completed. Considering the compromised immune function of the patient due to diabetes mellitus and the absence of recent hospitalization, a community-acquired infection was evident. Initial gastrointestinal symptoms suggested a possible bloodstream infection caused by negative Enterobacteriaceae, with *S. aureus* not being ruled out. Considering the potential renal impairment, empirical antibiotic therapy with imipenem/cilastatin combined with tigecycline was initiated, along with organ function support treatment. Twenty hours post-admission, the mNGS of blood samples revealed a high sequence count of *K. pneumoniae*, which was not antibiotic-resistant. At 72 hours, blood culture results indicated high virulence *K. pneumoniae*. As no definitive source of infection was found, the diagnosis was primary community-acquired Gram-negative rod (G-) bacteremia, resulting in the discontinuation of tigecycline medication. After one week of treatment, the patient’s peak body temperature decreased, and circulation stabilized, infection indicators improved, and subsequent blood cultures were negative. In accordance with the susceptibility test results, antibiotic therapy was adjusted to piperacillin/tazobactam. During treatment, the patient developed progressive deterioration of dry gangrene in the extremities of the limb (**Figure 1**). Vascular ultrasound revealed multiple arterial plaques and stenosis. After consulting with a specialist in vascular surgery, the patient received anticoagulant medication, papaverine to facilitate blood vessel dilation, and measures to prevent local infection. These interventions resulted in only partial improvement with a subsequent risk of amputation. Three weeks after treatment, the patient’s vital signs stabilized, renal function recovered, and he was transferred to a secondary-care hospital.

**Figure 2:**
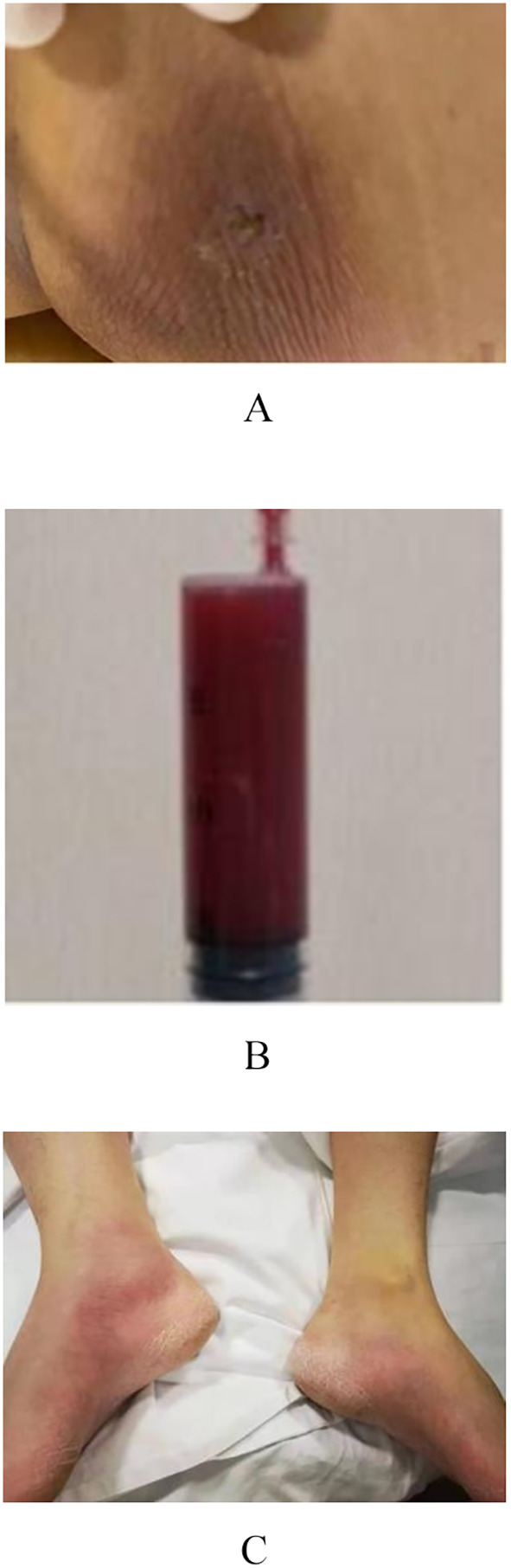
**(A)** Bacterial migration leads to the formation of buttock abscess. **(B)** The syringe contains puncture fluid for buttock abscess. **(C)** Symptoms of skin infection on both feet.

**Figure 3:**
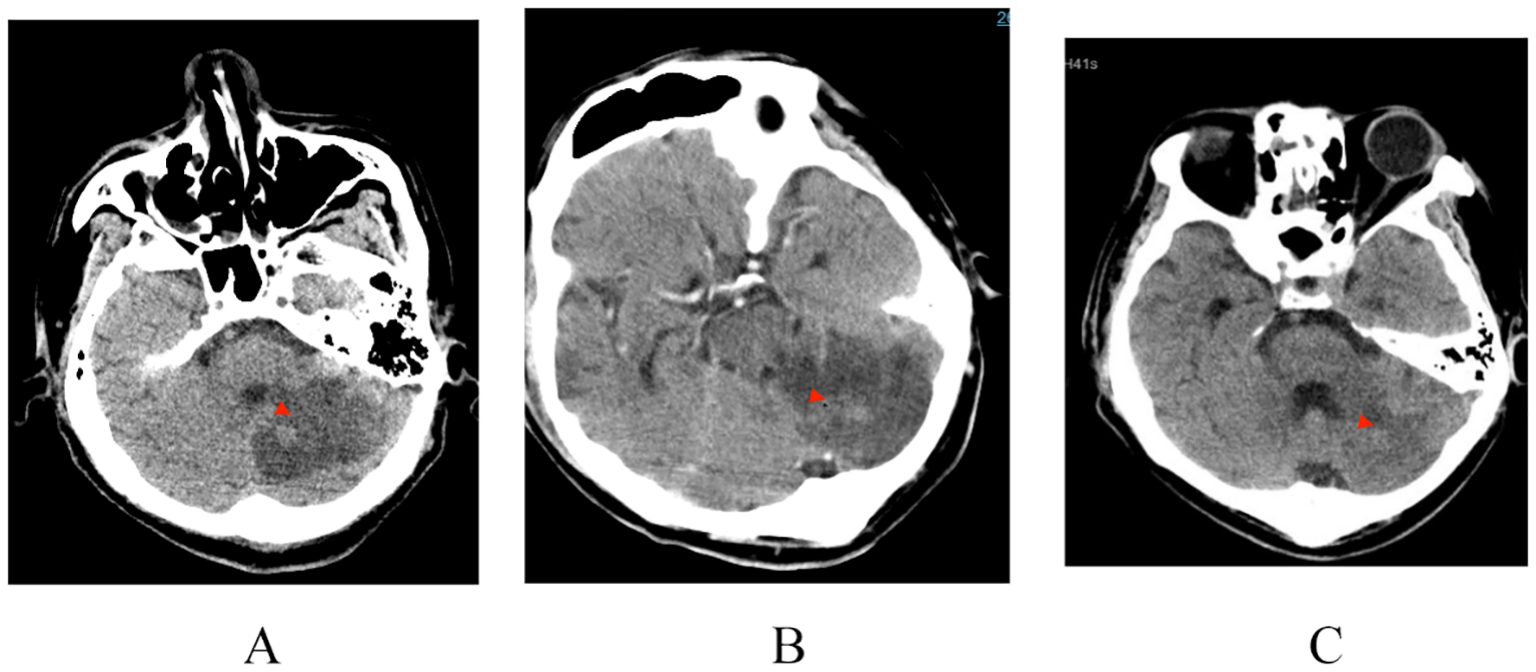
Bacteria migrate to intracranial abscess formation, gradually absorbed and improved after anti infection treatment. Cranial CT scans dated on **(A)** April 20 **(B)** April 26 **(C)** May 6.

**Figure 4:**
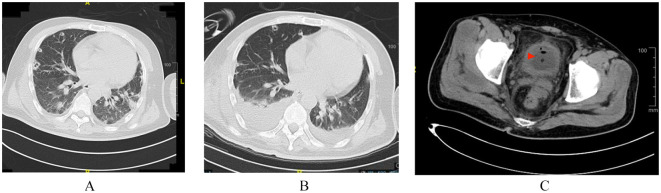
Bacteria migrate and spread into the lungs; Chest CT **(A)** April 18 **(B)** May 11; Formation of rectal bladder abscess. Pelvic CT. **(C)** April 19.

Patient-2: A 38-year-old male patient presented to the hospital with a four day history of high fever, altered mental status, and impaired right-sided limb movement, with a score of 20 on the APACHE II scale. His history of diabetes mellitus was unclear, with a glaciated hemoglobin level of 12.5%. Upon admission, his body temperature was 39°C, heart rate: 132 beats/minute, blood pressure: 75/40 mmHg, and respiratory rate: 40 breaths/minute. Physical examination revealed wet rales in both lungs, swelling and redness of soft tissues in the right shoulder and buttocks, increased temperature, and a sensation of tension. After puncture, bloody cloudy fluid was extracted, accompanied by cyanosis in both feet (**Figure 2**). Laboratory tests revealed WBC 40.85×10⁹/L, CRP 343.15 mg/L, PCT 3.1 ng/ml, and IL-6 1910 pg/ml. CT scans revealed a low-density lesion in the left cerebellar occipital region, scattered punctuate densities in the brainstem (**Figure 3**), multiple thick-walled cavities near the pleura in both lungs, and a fluid-like dark area near the rectum (**Figure 4**), and a fluid-like dark area near the rectum. Post-admission, blood, bronchoalveolar lavage fluid, and pus (from per rectal abscess) cultures, along with mNGS testing, were conducted. The diagnosis was community-acquired infection complicated by sepsis and septic shock. Subsequent investigation into the patient’s medical history uncovered a pattern of recurrent enema use for constipation four days prior. This practice resulted in episodes of bleeding and injury to the rectal mucosa, culminating in the formation of a local abscess and subsequent hematogenous dissemination to the brain, lungs, and soft tissues of the skin. The patient presented with pronounced clinical infection and toxic symptoms as a result of these complications. The initial antibiotic regimen consisted of the combination of vancomycin, piperacillin/tazobactam, and micafungin for empirical antibiotic treatment. Twelve hours post-admission, the laboratory conveyed findings of Gram-positive cocci observed in the aerobic bottle of the peripheral blood culture. At about 20 hours, mNGS results indicated high sequence counts of *S. aureus* in blood, bronchoalveolar lavage fluid, and pus. Following a consultation with an infectious disease specialist, the antibiotic regimen was changed to vancomycin combined with linezolid, and micafungin was discontinued due to the lack of definitive evidence of fungal infection. A surgical exploration of the pelvic abscess was performed, revealing a lateral rectal wall perforation and a rectovesical space abscess. Adequate debridement and ileostomy were performed. Transesophageal echocardiography was conducted to rule out the possibility of cardiac valve vegetation. A week subsequent to admission, the patient’s circulatory status gradually stabilized, prompting the discontinuation of vasoactive medications, and timely removal of deep venous and PICCO catheters. Concurrently, renal function improved, allowing for the cessation of continuous renal replacement therapy (CRRT). Daily assessments of consciousness consistently indicated a sustained altered level of consciousness, as evidenced by a Glasgow Coma Scale (GCS) score of E4VTM4 and the presence of right-sided hemiparesis. Subsequent cranial CT scans revealed a noteworthy reduction in the size of the left occipital low-density lesion, signifying the efficacy of the administered treatment. Two weeks after the admission, a percutaneous tracheostomy under the guidance of bronchoscope was performed, followed by weaning exercises. Two weeks later, the patient still experienced intermittent fevers around 38°C, with white blood cells maintained at 16×10⁹/L. Three repeat blood cultures were negative. Chest CT scans showed substantial absorption of the pulmonary infiltrates and a slight reduction in cavities. Abdominopelvic CT scans showed no significant fluid accumulation. However, the erythema and swelling of the soft tissue in the right shoulder and buttock had increased, with CT scans revealing localized fluid-like dark areas. Ultrasound-guided puncture and drainage were actively performed, resulting in the resolution of fever. The antibiotic regimen was modified to cefuroxime in combination with linezolid. Three weeks after admission, the patient’s vital signs stabilized, and there was a marginal improvement in consciousness. Consequently, the patient was transferred to a rehabilitation hospital for further therapeutic intervention.

## Discussion

The incidence and mortality rates of CABSI are on the rise globally (15). According to studies, pre-hospital delays and inappropriate empirical antibiotic treatment are significant risk factors for an increased risk of fatality within 30 days for patients with CABSI (2, 6). The timely administration of antibiotic treatment, guided by clinical evidence, is imperative. Nevertheless, inappropriate or prolonged use of empirical antibiotic medications can lead to the overuse of antibiotics and the emergence of antibiotic resistance (16). The prompt acquisition of etiological evidence utilizing available diagnostic techniques can inform the judicious use of antibiotics, thereby reducing mortality and disability rates while minimizing the risk of antibiotic resistance. Conventional laboratory testing methods(For example, bacterial culture) have longer turnaround times and suffer from poor sensitivity and specificity, often being influenced by antimicrobial drugs, thus failing to provide early clinical assistance. PCR care point (POC) is a molecular biological technique that can be performed at the bedside to rapidly amplify specific DNA sequences, and the results can usually be obtained within a few hours. However, it is only used to detect known specific pathogens, and can only be used for qualitative detection, not quantitative detection.mNGS is an innovative nucleic acid detection technology based on high-throughput sequencing, which can simultaneously sequence all DNA or RNA in clinical samples, especially for unknown and difficult-to-detect pathogens, as well as critical mixed infections. This method has the characteristics of comprehensive coverage, unbiasedness, short detection cycle, high positive rate and strong specificity. It is suitable for all kinds of clinical specimens, can be qualitative and quantitative at the same time, and can be used for drug resistance analysis. It has greater guiding significance for clinical diagnosis and treatment. Compared with other detection techniques, it has unparalleled advantages (17). Both individuals discussed in this article were diagnosed with CABSI utilizing the mNGS technique. Blood, being inherently a sterile body fluid, may typically exhibit low concentrations of microbial nucleic acids even in the absence of viable microorganisms. This characteristic renders blood samples particularly valuable for diagnostic mNGS. Particularly for individuals in the ICU grappling with life-threatening severe infections, it is advisable to promptly conduct mNGS testing upon admission to enhance prognosis.

The two patients described in this article were both diagnosed with CABSI. Common clinical features in both cases included varying degrees of immunocompromised status, insidious onset with delayed medical consultation, and a suspected gastrointestinal origin of infection. Both patients initially received appropriate antibiotic treatment and underwent prompt completion of mNGS testing. Within 24 hours, the nature of the infections was swiftly elucidated, facilitating specific sequential treatment. A multidisciplinary collaborative approach involving specialists in infectious disease, gastroenterology surgery, vascular surgery, and other relevant clinical departments was implemented resulting in favorable outcomes. This approach helped to avoid the risks associated with antibiotic misuse, such as antibiotic resistance and adverse reactions, and reduced the cost burden. This demonstrates the application value of mNGS technology in the context of CABSI. In patient 1, blood mNGS results showed high virulence of *K. pneumoniae*, a member of the Enterobacteriaceae family known for its strong resistance to external factors and propensity for antibiotic resistance to most antibiotics. This pathogen is commonly found in the gastrointestinal and respiratory tracts. In this case, the patient was infected with a sensitive strain of *K. pneumoniae* originating from the gastrointestinal tract, which contributed to a relatively better prognosis.

In patient 2, mNGS results identified *S. aureus* as the etiological agent. The clinical presentation involved migratory changes in multiple sites, including the formation of multiple cavities in the lungs, skin and soft tissue infections, and brain abscesses. This was considered to be due to bacterial dissemination into the bloodstream following gastrointestinal injury, with rapid progression of the disease. In addition to the combined administration of vancomycin and linezolid for anti-staphylococcal treatment targeting different infection sites, aggressive surgical debridement formed the basis of effective anti-infective therapy. Beyond effective anti-infective treatment, a comprehensive and detailed medical history is crucial in determining the origin and characteristics of the infection. There is a need to strengthen pre-hospital and emergency department triage capacities, including training at community healthcare institutions and improvements in emergency triage systems. These measures can reduce pre-hospital delay for patients with CABSI, facilitate early identification of the risk of progression to sepsis, and thus improve survival rates. Both patients experienced prolonged pre-hospital delays due to inadequate diagnosis of their underlying conditions and the severity of their illness, which subsequently complicated later treatment efforts. Therefore, supervised education should be provided to patients with chronic diseases in long-term outpatient care to enhance their awareness of the requirement for timely medical consultation and follow-up, thereby minimizing the impact of delayed pre-hospital medical consultations on treatment outcomes. Insufficient treatment duration of bloodstream infections, caused by the lack of accurate indicators to guide the duration of anti-infective treatment, can lead to the recurrence or persistence of infections. mNGS-guided anti-infective therapy enables the monitoring of changes in the sequence count of the pathogen, enabling evaluation of the effectiveness of the treatment and facilitating rational medication use throughout the course of treatment. Currently, due to limitations in mNGS testing equipment and its relatively high cost, mNGS has not yet become a primary clinical testing technique, and delays in testing can limit its prognostic utility. It is advisable for patients with severe infectious diseases in the ICU to communicate the necessity of mNGS testing with their families and to conduct the testing as early as possible to obtain microbial evidence. Interpretation of mNGS results should be integrated with clinical and other laboratory findings. A multidisciplinary collaborative approach is recommended to ensure the accuracy of the results, avoid indiscriminate use of antibiotics based solely on these results, and facilitate effective antibiotic management.

During the whole treatment process, both patients showed different degrees of limb peripheral cyanosis after the application of vasoactive drugs. The basic vascular conditions should be considered when selecting the type and dose of vasopressors, and the occurrence of ischemic events should be alerted.

## Conclusion

The purpose of this study was to emphasize the increase in morbidity and mortality associated with CABSI by analyzing 2 severe cases of CABSI, with particular attention to immunodeficiency patients. These two cases highlight the importance of reducing pre-hospital delays, early identification of sepsis risk, a comprehensive and detailed understanding of the patient ‘s medical history and appropriate empirical antibiotic treatment. It is suggested that mNGS detection should be completed early to guide precise targeted therapy, which is helpful to guide rational drug use and improve prognosis. It is recommended to be used clinically.

## Data Availability

The original contributions presented in the study are included in the article/supplementary material. Further inquiries can be directed to the corresponding author.
